# Edible cellulose-based colorimetric timer†

**DOI:** 10.1039/d3nh00006k

**Published:** 2023-03-31

**Authors:** Gen Kamita, Silvia Vignolini, Ahu Gümrah Dumanli

**Affiliations:** a Department of Chemistry, University of Cambridge Lensfield Road Cambridge CB2 1EW UK; b The School of Materials, The University of Manchester Oxford Road Manchester M13 9PL UK ahugumrah.parry@manchester.ac.uk; c Henry Royce Institute, The University of Manchester Oxford Rd Manchester M13 9PL UK

## Abstract

A biocompatible and edible colorimetric timer is obtained by exploiting the dynamic colour changes of the cholesteric liquid crystalline mesophases of hydroxypropyl cellulose (HPC) in aqueous suspensions. The edible timer is encapsulated between semi permeable membranes made of shellac. The cholesteric organisation of the HPC provides vibrant colouration, while the shellac layers allow tuning of the evaporation rate of water from the mesophase, which results in a colour change. Due to the biocompatibility of the components and the direct read-out of the system, *i.e.* the colour change can be visually detected, and the developed timer can be implemented as a colorimetric sensor with potential to be used in food packaging, and as a smart labelling system.

## Abstract 2

A biocompatible and edible colorimetric timer is obtained by exploiting the dynamic colour changes of the cholesteric liquid crystalline mesophases of hydroxypropyl cellulose (HPC) in aqueous suspensions.

## Graphical abstract

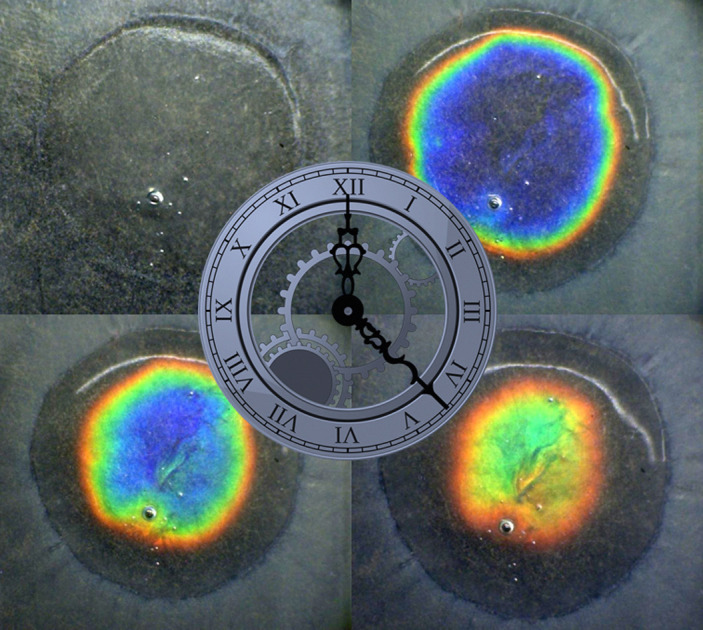

**New concepts**

Formation of lyotropic phases of cellulosic polymers was first demonstrated in the 1970s. However, the use of cellulose based liquid crystals in optical devices has been limited due to their lack of colour response for electric fields. Therefore, investigation of HPC-based colour changing technologies and displays is still in its infancy. Our work demonstrates a simple concept through coating the HPC liquid crystalline phase with a semi-permeable and transparent shellac film, which controls the rate of the colour change by modulating the water absorption and evaporation rate from the liquid crystalline phase. The thickness of the shellac layer is the key parameter to finely-tune the timer function of this material based device. This work is the first to exploit the dynamic colour changes of HPC and formulate a multi layered colorimetric device, together with achieving a thorough understanding on the optical properties of this system. The dynamic reversibility of the coloration and the direct colour read-out from these systems will open new horizons in terms of manufacturing colorimetric devices from mesophases of cellulose, targeting applications in food and pharmaceutical packaging applications.

## Introduction

**Fig. 1:**
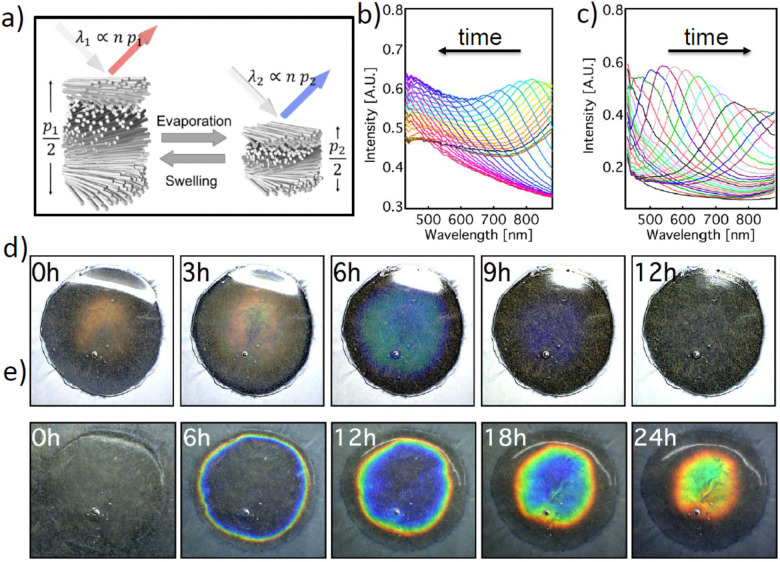
(a) Scheme of the cholesteric HPC mesophase, with pitch (*p*) describing a 360° helicoidal rotation of the polymeric chains and reflection colour changing as a function of the drying and swelling processes. (b) diffuse reflection spectra of the HPC coating during drying and (c) Diffuse reflection spectra of the HPC coating during swelling. (d) and (e) Photographs of the HPC edible colour timer film system during drying and swelling, respectively.

Hydroxypropyl cellulose (HPC) is a water-soluble cellulose derivative that has been used as an emulsifier, stabilizing agent and thickener for the food industry^1^ due to its viscoelastic behaviour and biocompatibility.^2^ HPC is also used as a film forming agent and an inactive ingredient in the pharmaceutical industry for providing sustained-release of drugs.^3,4^ While the use of HPC in the pharmaceutical industry sustains the bulk production of this polymer, HPC is also a functional polymer that spontaneously self-assembles into the cholesteric liquid crystalline phase in a variety of solvents including water and displays lyotropic and thermotropic liquid crystalline properties.^5–7^ This cholesteric phase interacts with light, providing a strong intense colouration when the cholesteric pitch is comparable with the wavelengths of visible light, see Fig. 1a. The colouration change is a function of the relative concentration of water and HPC.^8,9^ Many efforts have been dedicated to understanding the colour formation dynamics of lyotropic HPC in a sealed liquid phase^8,10^ or retaining colour in solid form *via* the addition of a cross-linking agent^7,11^ or incorporation of other cholesteric derivatives.^12,13^ However, the main idea presented in this study, the dynamic and reversible behaviour of cholesteric HPC as a colour shifting functional soft matter system, has not been explored thus far.

In this work, we utilized the dynamic liquid interactions of the HPC mesophase in swelling and drying conditions controlled by applying a semi-permeable coating to build a colorimetric timer. Basically, in the timer construct, the HPC gels formed a uniform cholesteric phase that was encapsulated inside a transparent and semi-permeable film made of shellac. The semi-permeable layer allowed tuning of the evaporation rate in a reversible manner by controlling the water absorption and evaporation as a function of thickness, *i.e.* the evaporation kinetics can be directly converted into a time scale to use this system as a colorimetric timer. As the colouration of the HPC mesophase was dependent on the water content, the water loss induced a blue-shift in the reflection wavelength. Inducing such a colour shift enabled us to develop an edible colorimetric timer in which the colour shift rate can be adjusted from a few minutes to several hours. A crucial feature of this colorimetric timer system is the complete reversibility of the colour change process, and the system could in fact be re-hydrated by simply immersing it in water and reused. We believe that the developed system offers a safe and multifunctional colorimetric timer coating solution that is easy to fabricate and monitor. We envisage that such a functional system with a reversible and visual response to changes in the external environment is ideal for monitoring food freshness, as it does not require any electronic apparatus, which is often incompatible, non-practical or costly for food packing applications.

## Experimental

### Materials preparation

Highly concentrated HPC suspensions (60%/wt) were prepared by mixing HPC powder (MWav 100 000 g mol^−1^, available from Sigma-Aldrich UK) with DI water. The solution at this concentration forms a highly viscous gel. Due to the long relaxation time of the HPC polymer chain, the mesophase was kept for a few weeks in a sealed container and occasionally mixed with a spatula to reach a homogeneous cholesteric phase. After the HPC cholesteric phase reached equilibrium and showed homogeneous colouration, it was spread onto a transparent disk-shaped container. To obtain a flat interface and remove any excess HPC mesophase, the disk container was then pressed against a parafilm sheet mounted on a rigid support. At this point, the HPC that was sandwiched between 2 glass slides was frozen by immersing it into liquid nitrogen for a few seconds and the rigid parafilm support was quickly removed leaving a flat top surface. The sample was then dried by heating it to 50 °C with a hotplate overnight and was weighed. The dimensions of the dry HPC film were about 1.5 cm in diameter and 0.5 mm in thickness and it typically weighed 90 mg.

The dry films were subsequently encapsulated with shellac *via* spin coating. Shellac solutions with various concentrations ranging between 5% and 10% by weight in *n*-isopropanol were prepared to vary the thickness of the coating layer. After a spin-coating step, the shellac layer was dried by keeping the sample on a hotplate at 50 °C for about 1 hour. The sample was weighed again and the weight of shellac was calculated by subtracting the weight of HPC and the glass slide.

### Spectroscopic measurements

The scattered reflection spectra of HPC during the swelling/drying processes were recorded with a Bifurcated fibre probe attached to a spectrometer (Ocean Optics QE65000). A reference was taken with a Lambertian diffuser. The swelling of the HPC/shellac film system was achieved by immersing the sample into a beaker with DI water. The fibre probe was submerged into the water at the centre of the film and angled against the sample normal by 10–20° in order to collect the scattered reflection of HPC only and not the specular reflection of the sample surface. After reaching sufficient swelling, the sample was taken out of the beaker and the water on its surface was removed. The sample was dried under ambient conditions (at room temperature and 50% humidity) while placed on a balance and was weighed periodically with a computer connected to the balance, which had a Python program that sent instructions to the balance by serial communication. At the same time, the scattered reflection was measured with a fibre probe as before, with the same tilt angle against the sample normal as before.

### Estimation of the shellac layer thickness

The thickness of the shellac coating was measured by analysing the results of reflectometry measurements. The shellac coating was removed from used samples by immersing them in water until the HPC disc was completely dissolved. The remaining shellac film was transferred onto silicon chips and their reflection spectra were recorded using micro-spectroscopy with an optical microscope (Olympus BX-51).^14^ Assuming a refractive index of shellac of *n* = 1.52,^15^ the interference fringes were modelled with an automated film thickness analysis program written in Igor Pro.^16^

### The microstructural analysis

The thickness of the shellac layers was confirmed by cross sectional SEM. Prior to the SEM imaging, the timer samples were fully dried at 50 °C in a convection oven for 4 hours. The dried HPC–shellac films were scratched in the middle with a razor and fractured completely to capture the cross-section morphology. These fractured samples were fixed at a 90 degree angle to the sample holder using thin copper tape and Au/Pt was deposited onto the samples using a Quorum sputter-coater Q150R Plus prior to imaging (an 80 : 20 Au/Pt target was used at 50 mA current for 8 seconds). The cross-sectional microstructure was analysed using a Zeiss Sigma VP FEG SEM system. The imaging was done at 3 keV accelerating voltage and at a 2.9 mm working distance.

## Results and discussion

We investigated the colorimetric response of the HPC gel systems that were encapsulated in a semi-permeable shellac layer under drying and re-hydration conditions. Depending on the average molecular weight, above a critical concentration (about 40–48% wt), HPC and its derivatives form cholesteric mesophases in water and in a wide range of organic solvents.^7,17^ The materials organization in such cholesteric mesophases can be visualized as a stack of helicoidal quasi-layers yet the polymers possess degrees of freedom to move dynamically to fold, deform and adapt different orientations due to the presence of water. In these cholesteric phases, the full distance of the helicoid, cholesteric pitch *p* and the average refractive index *n* define the colour of the macroscopic material,^18^ as schematically depicted in Fig. 1a. At equilibrium, the value of *p* is a function of HPC concentration in water, whereas *n*_*av*_ could also dynamically change as the HPC:water ratio changes; *i.e.*, 60% wt HPC would have an *n*_av_ value of 1.456 and 70% wt HPC would have an *n*_av_ value of 1.464 (using Eqn 1 and assuming a refractive index of HPC of 1.52^19^).

1 *n*_av_ = *n*_HPC_*ϕ*_HPC_ + *n*_Water_*ϕ*_Water_

Thus, the effect of the average refractive index on the change of colour in this concentration range can be considered to be relatively small. The direct relation of the reflective wavelength to the refractive index and the pitch can be expressed as a modified Bragg equation, see eqn (2); the colour of the mesophase therefore provides a direct colour reading to monitor the dehydration state of the system.

2 *λ* = n.p. cos*θ*

The cholesteric pitch of lyotropic HPC is a function of concentration and the molecular weight of HPC even with a 1% loss of water can cause a significant change in the pitch.^7^ Therefore, during the evaporation and water absorption processes, the pitch (*p*) of the HPC mesophase changes significantly. While the water evaporation process causes a blue-shift to shorter wavelengths, the re-hydration process shifts the pitch (colour response) back to the initial position. Such a time dependent colorimetric response can be quantitatively monitored spectroscopically using a bifurcated fibre probe measuring only the signal reflected from the central area of the system, as depicted in Fig. 1b and c. The visible colour change during the swelling and drying configurations can also be observed, as shown in Fig. 1d and e.

The HPC gels were cast on substrates using an injector to obtain a round shape. The gels then formed a water droplet shape with a parabolic profile upon coating with the shellac layer and the final HPC constructs kept this parabolic profile, see Fig. S1a–c, ESI.† Such a parabolic shape in the HPC constructs correlates to a thickness change of the HPC throughout the sample, which induces a colour gradient for both water evaporation and absorption interactions, as shown in Fig. S1b; ESI.† The parabolic shape of the timer and the thickness gradient also affected the kinetics of the water evaporation and absorption processes; *i.e.*, the water absorption into the HPC phase was not uniform within the entire area of the sample and the round droplets of the HPC gels appeared to possess concentric colour patterns in the timer device, see Fig. 1e. It is also worth noting that the water evaporation and absorption kinetics in these constructs differ, as evidenced by the colour change observed over 12 hours under evaporation conditions and the same construct taking 24 hours to show a similar colour change for water absorption. Such a difference in the kinetics can be attributed to the different diffusion dynamics between the hydrated and de-hydrated phases, *i.e.* the dehydrated phase consists of collapsed cholesteric layers, which have slower kinetics to diffuse water compared to the open layered structure in the hydrated phase. However, the presented samples of the colorimetric devices studied in this work demonstrated a colour shift variation across the samples. This effect can be minimized by increasing the sample area and changing the mechanical stability of the membrane in a scaled-up production set-up.

**Fig. 2:**
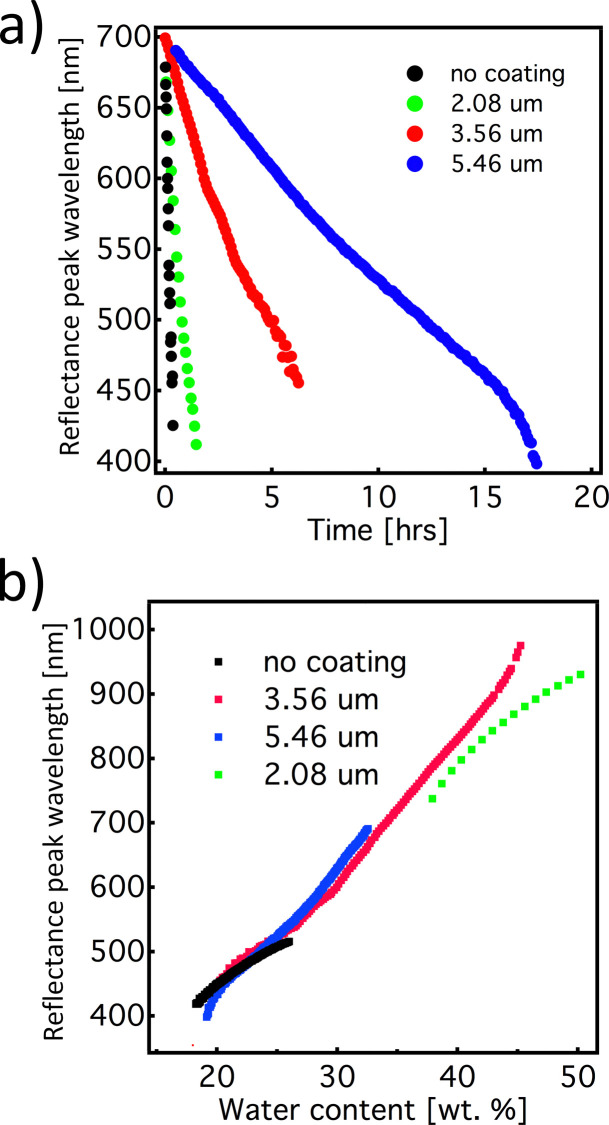
. (a) Kinetics of the reflection peak wavelength as a function of shellac layer thickness upon drying. (b) Reflection peak wavelength as a function of HPC/water ratio for samples with different shellac coating thicknesses. The colours shown in the graphs represent different thicknesses of the shellac layer: no coating (black), 2.08 um-thickness (green), 3.56 um-thickness (red), and 5.46 um-thickness (blue).

The time-response of the system can be readily tuned by simply adjusting the thickness of the semi-permeable shellac layer. The graphs in Fig. 2a and b show the response of the mesophase for different thicknesses of the shellac coating. As expected, we observed that by increasing the thickness of the coating, the evaporation time increased. Interestingly, the rate of the colour change, during both the swelling and the drying processes, shows linear behaviour, regardless of the thickness of the shellac coating. Furthermore, if wavelength of the reflection peak is plotted as a function of the HPC mesophase concentration, the data points corresponding to different samples are aligned with a small discrepancy, indicating that the spectral information provides an excellent measurement of the water content in the mesophase. This attribute makes this timer system quite robust in the sense that the shellac coating thickness, without influencing other properties, can adjust the colour-shift rate. This allows great flexibility in terms of colour tuning for specific functions, *i.e.* coupling the colour change to a drug release system or to predict humidity related freshness for perishable food items.

**Fig. 3:**
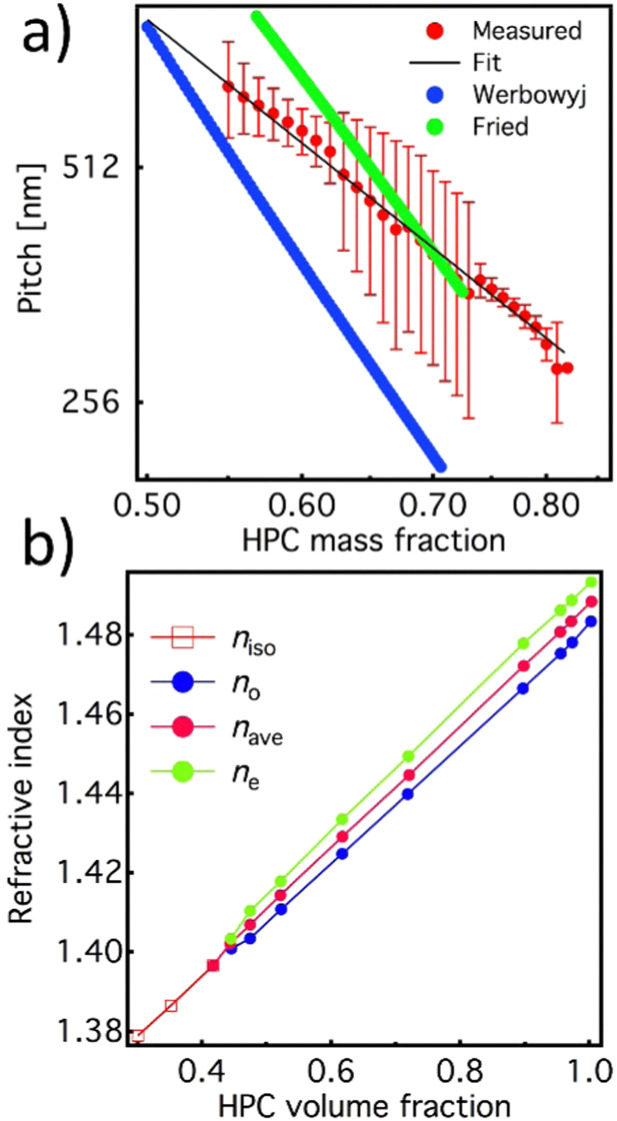
(a) Extrapolated cholesteric pitch values (red line: experiment and black line: fit) as a function of the HPC/water ratio acquired with the balance and compared with the literature values from Werbowyj^5^ (blue line) and Fried^20^ (green line). (b) Value of the average refractive index (red) of the HPC mesophase extrapolated from ordinary and extraordinary values of refractive index calculated using eqn (1).

The reproducibility of the developed system allowed us to use it to further characterize the dimension of the cholesteric pitch as a function of the relative HPC/water ratio and compare it to the ones reported by Werbowyj^5^ and Fried.^20^ To do so, we used the de Vries formula^18^ using as average refractive index the one reported in Fig. 3a measured by Werbowyj^5^ and considering the effective medium approximation given in eqn (1), and using as refractive index the one reported in Fig. 3b measured by Onogi *et al*.^19^

The relation between pitch and polymer concentration can be expressed as the following empirical relation:^21^3
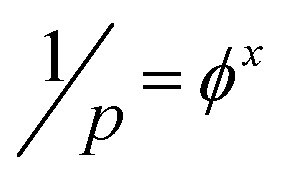
where the volume fraction *ϕ* can dictate the pitch value, where *x* is between 2 and 4. Kimura *et al.*,^22^ on the other hand, extended this relationship by accounting for the effect of temperature. Therefore, they predicted a linear dependence of the reciprocal pitch expressed as4
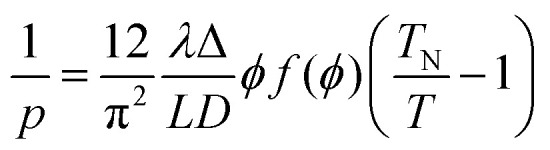
where

5 *f*(*ϕ*) = [1 − (*ϕ*/3)]/[1 − *ϕ*]^2^

where *ϕ* is the volume fraction of particles with a length *L* and diameter *D*, *λ* and Δ are factors that characterize the helical shape of the molecules, *T* is the actual temperature of the system, and *T*_N_ is the temperature at which the twist disappears as the pitch approaches infinity, *p* → ∞. The cholesteric pitch values obtained in this work agree with this linear relationship and the existing work of Fried^20^ but are significantly different from the ones reported by Werbowyj.^5^ The discrepancy between these studies can be explained by the sample handling temperatures,^23^ imperfections in the homogeneity of the mesophase, see the ESI,† and most likely by the differences in molecular weight of the starting HPC polymers. It is also worth noting that in the compared work by Fried and Werbowyj, the HPC solutions were individually prepared at the desired weight percentages and measured in equilibrium in a closed system and therefore can be considered as static. In our work, however, the water absorption and evaporation mechanisms cause a dynamic change in the colour. At the same time, the overlap between the fit and the pitch values confirms that the dynamic colour changes we observed indeed relate to the polymer:water ratio of the liquid crystalline phase at equilibrium.

## Conclusions

In conclusion, we demonstrate the fabrication of a colorimetric timer system based on encapsulation of a HPC cholesteric mesophase into water permeable membranes consisting of shellac. The mechanism of the colour change as a function of HPC–water content was investigated in detail by studying the system during swelling and drying processes. The time response can be easily monitored as a colour change and tuned by simply changing the shellac coating thickness. The biocompatibility, availability and responsiveness of the materials used in the system make it particularly suitable for applications in smart packaging and labelling.

## Author contributions

The HPC mesophase formulations, study of phase behaviour and optimisation of the bilayer constructs were conducted by AGD and GK. The optical microscopy and spectral analysis of the colorimetric timers were performed by GK. The SEM images were captured and analysed by AGD. The spectral data analysis and first drafting of the manuscript were done by GK, SV and AGD. All of the authors reviewed the manuscript and were involved in the further iterations of the work and AGD produced the final version of the manuscript.

## Conflicts of interest

There are no conflicts to declare.

## Supplementary Material

NH-008-D3NH00006K-s001
