# Supplementary material for: Edible cellulose-based colorimetric timer
Source: Nanoscale Horiz. 2023 Mar 31;8(7):887–91. doi: 10.1039/d3nh00006k (PMC10291961; doi:10.1039/d3nh00006k)
Supplement: NH-008-D3NH00006K-s001 [file NH-008-D3NH00006K-s001.pdf]

Electronic Supplementary Material (ESI) for Nanoscale Horizons.
This journal is © The Royal Society of Chemistry 2023

Supporting Information

Edible cellulose-based colorimetric timer†

Gen Kamita[a], Silvia Vignolini*[a] , and Ahu Gümrah Dumanli*[a,b,c]

a. Department of Chemistry, University of Cambridge, Lensfield Road, Cambridge CB2 1EW UK
b. The School of Materials, The University of Manchester, Oxford Road. Manchester M13 9PL UK
c. Henry Royce Institute, The University of Manchester, Oxford Rd, Manchester, M13 9PL, UK

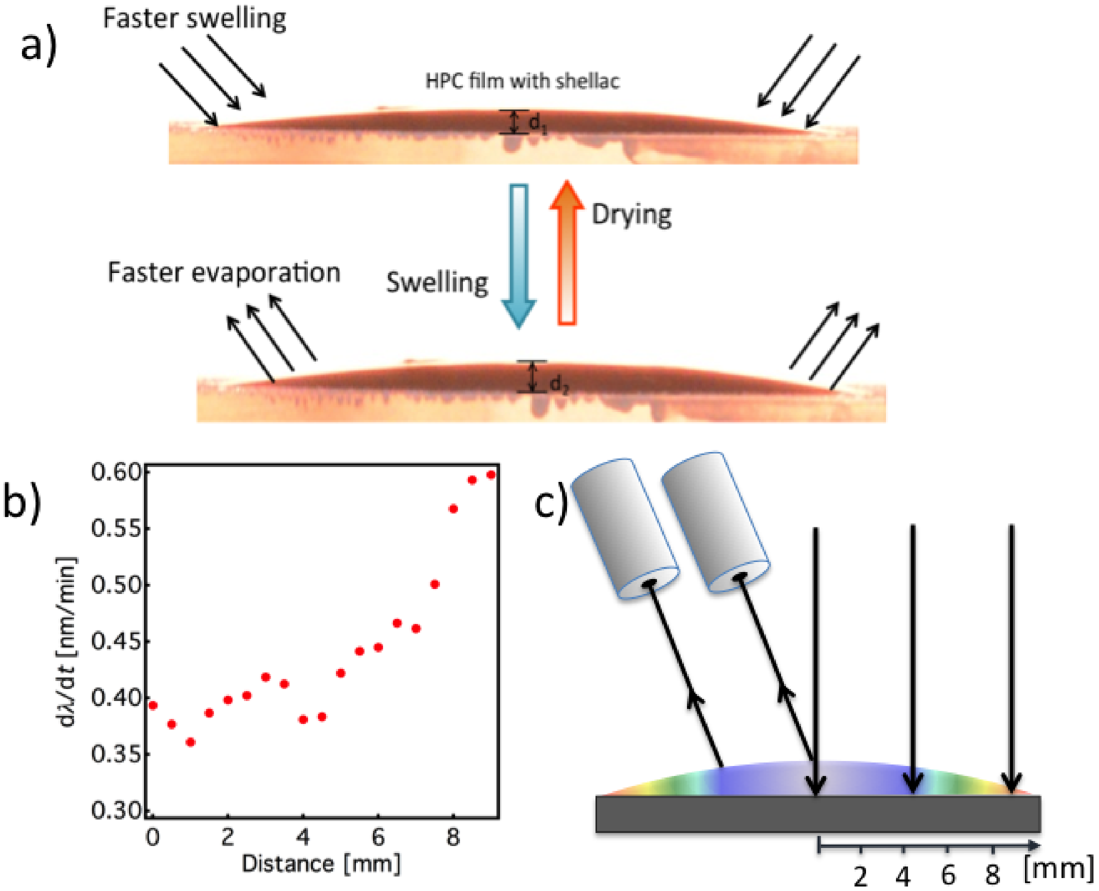

Figure S1. a) Photographic image of the side view of the HPC with shellac timer and the changes in the thickness as a function of water evaporation and swelling b) The colour shift of the timer device as a function of distance from the centre of the device construct in mm. c) Schematic representation of the colour shift experiment presented in (b)

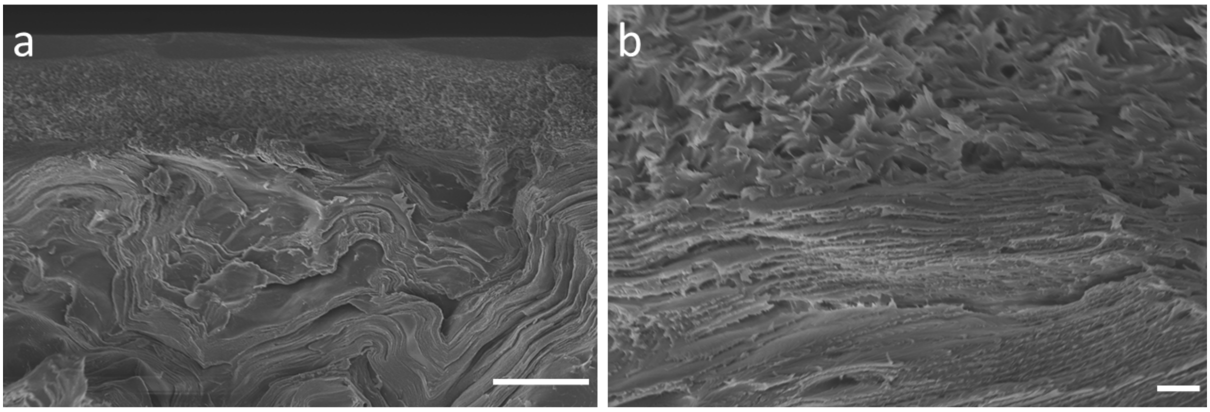

Figure S2. Scanning electron micrographs of the HPC-Shellac timer device in cross-sectional configuration in dry state demonstrating the cholesteric phase of the HPC and the semi-permeable network of the shellac demonstrating no order that can interfere with the light reflection from the HPC cholesteric phase. The scale bars are **a)** 5 µm and **b)** 1 µm
